# Non-Invasive Paraclinical Diagnosis of Hepatocutaneous Syndrome in a Dog

**DOI:** 10.3390/life14070853

**Published:** 2024-07-08

**Authors:** Anamaria-Hortensia Strichea, Ștefania Livia Hreniuc, Gheorghe Solcan

**Affiliations:** 1 Internal Medicine Clinic, Faculty of Veterinary Medicine, Iași University of Life Sciences, 8 M. Sadoveanu Alley, 700489 Iași, Romania; anamaria.strichea@iuls.ro; 2 Diagnostic Imaging Clinic, Faculty of Veterinary Medicine, Iași University of Life Sciences, 8 M. Sadoveanu Alley, 700489 Iași, Romania; hreniucstefania@gmail.com

**Keywords:** hepatocutaneous syndrome, necrolytic migratory erythema, superficial necrolytic dermatitis

## Abstract

A 14-year-old, female spayed Bichon Maltese with no other known previous pathologies was presented for dermatological examination after being referred from a private clinic with the suspicion of generalized, treatment-resistant demodicosis. Upon presentation and clinical examination, multiple deep skin scrapings were performed, returning negative parasitological results. Complete blood count and serum biochemistry revealed mild hepatic damage. Abdominal ultrasound revealed an abnormal echostructure of the liver displaying a honeycomb or Swiss cheese-like pattern, reported as pathognomonic for hepatocutaneous syndrome. The owner declined any further paraclinical examination, including skin biopsy and histopathological examination, requesting a treatment protocol that could be pursued at home, considering the age of the dog and its reactive behavior during the examination. The present case report highlights a non-invasive method of diagnosing the hepatocutaneous syndrome in a dog by clinical examination, routine blood testing, and ultrasound assessment of the abdomen, in the absence of the possibility of doing a histopathological diagnosis.

## 1. Introduction

Superficial necrolytic dermatitis is an uncommon and frequently lethal condition in dogs, often linked to pancreatic neuroendocrine neoplasia and hepatocutaneous syndrome [1,2]. In human medicine, necrolytic migratory erythema is a term used to characterize the skin rash observed in individuals with a pancreatic tumor that secretes glucagon (glucagonoma) or occasionally in cases of hepatic cirrhosis and some gastrointestinal disorders. This rash has also been observed in dogs and cats, often in conjunction with liver disease. Other terms such as hepatocutaneous syndrome, superficial necrolytic dermatitis, and metabolic epidermal necrosis are frequently employed to refer to the same condition [3,4].

The precise pathogenesis remains uncertain, but it is believed that elevated gluconeogenesis due to hyperglucagonemia (associated with pancreatic tumors) or heightened hepatic breakdown of amino acids (in cases of chronic liver disease) leads to decreased plasma amino acid levels and depletion of epidermal proteins. This depletion is what causes the skin lesions characteristic of superficial necrolytic dermatitis. While uncommon in dogs and rare in cats, the condition is most frequently observed in older animals [5].

The lesions initially manifest in regions of the body with high cell turnover, such as mucocutaneous junctions, the face, the footpads, and areas prone to pressure. These lesions typically consist of crusts and ulcers accompanied by reddened skin surrounding the affected area. Additionally, most affected dogs exhibit non-regenerative anemia, mild elevation in blood sugar levels, heightened serum liver enzyme levels, and a liver appearance resembling a “honeycomb” pattern under abdominal ultrasonography [3,6,7,8]. Although there are some reports regarding concurrent pancreatic changes [9], they were not observed in the present case.

## 2. Case Description

A 14-year-old Bichon Maltese was presented for dermatological examination to the Internal Medicine Clinic of the Faculty of Veterinary Medicine of Iași. The dog had previously undergone examination and a treatment protocol for demodicosis at a private clinic. The treatment included three consecutive monthly external antiparasitic treatments: the first two with an oral formulation (Nexgard Spectra, Boehringer Ingelheim International GmbH, Ingelheim am Rhein, Germany) containing afoxolaner (37.50 mg)/milbemycin oxime (7.50 mg) as active substances, and the third with a spot-on application of an imidacloprid (100 mg)/permethrin (500 mg) external antiparasitic (Advantix, Elanco, IN, USA). Additionally, the dog had been bathed once a week for the previous four weeks using a sebolytic shampoo (Sebolytic, Virbac, Suite, TX, USA). The animal had not received any drugs potentially responsible for drug-induced skin lesions [3,5,6]. Despite the antiparasitic treatments and topical therapy, no positive results were observed, prompting a referral for a second opinion.

The owners reported that the dermatological condition had begun approximately three months prior to the examination, and although some lesions appeared to have healed, new lesions emerged in different areas. When questioned about pruritus, the owners rated it as 5/10 on the PVAS scale [10]. According to the owners, the dog had a good appetite and no other known health issues apart from the cutaneous lesions, which were observed to be painful as evidenced by increased sedentarism and evident mobility challenges attributable to inflammation affecting the paws. The dog’s diet consisted of a mixture of human food and commercial wet dog food.

**Figure 1:**
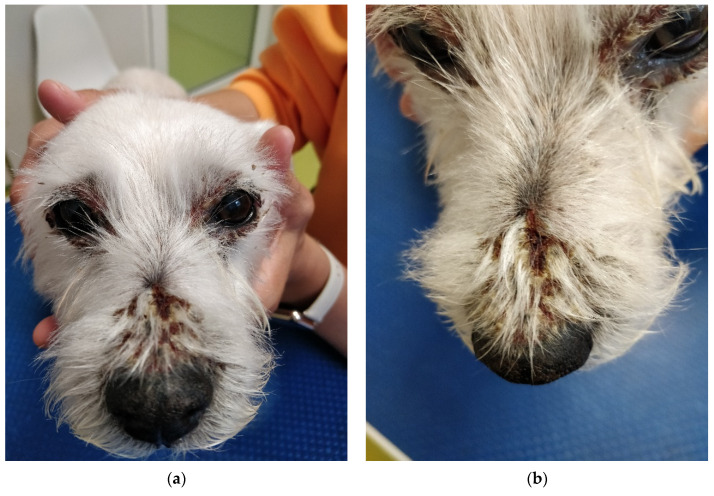
(**a**) Crusted lesions localized periocular and on the nasal planum. (**b**) Crusts situated on the nasal planum.

**Figure 2:**
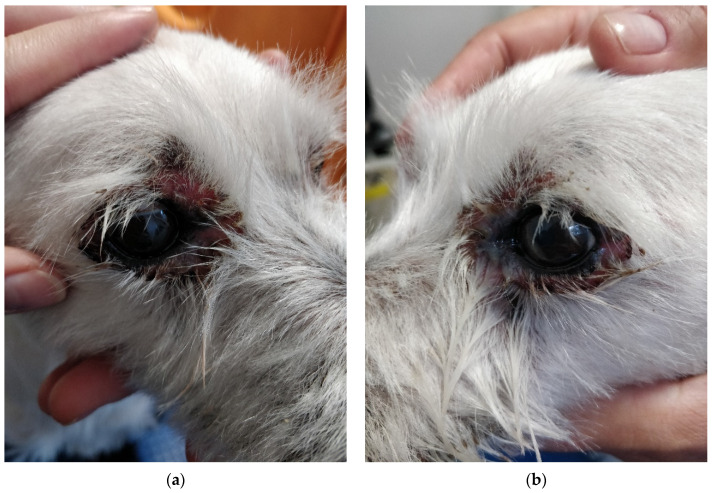
(**a**) Erosions in the periocular area of the right eye accompanied by adjacent alopecia. (**b**) Erosions in the periocular region of the left eye accompanied by adjacent alopecia and purulent discharge.

**Figure 3:**
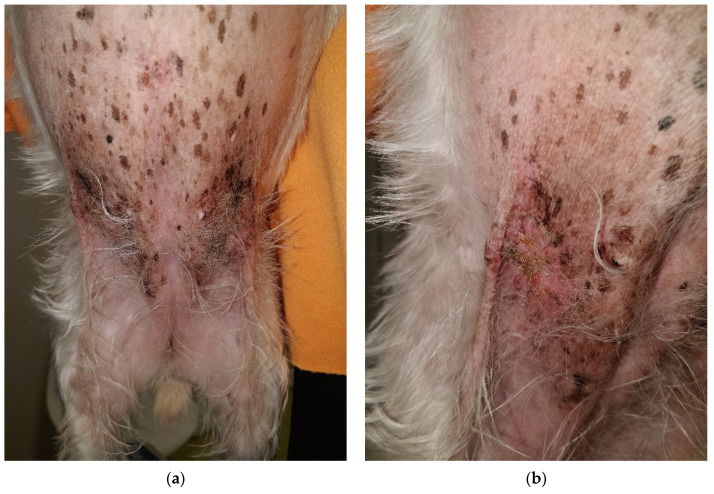
(**a**) Symmetrical appearance of the lesions situated in the inguinal region. (**b**) Ulcerated lesion covered with crust and adjacent erythema observed in the right inguinal area.

**Figure 4:**
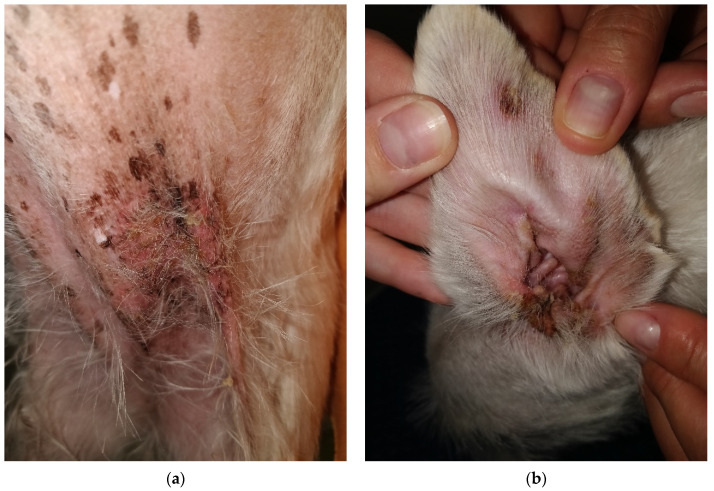
(**a**) Ulcerated lesion with erythema observed in the left inguinal area. (**b**) Round erythematous and crusty lesion located near the margin of the left pinna, along with the presence of erythema and crusts at the entrance of the ear canal.

**Figure 5:**
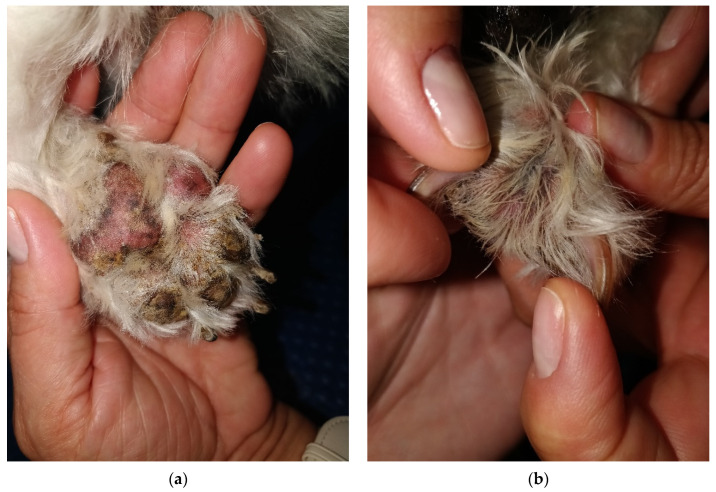
(**a**) Severe pododermatitis with the presence of purulent discharge and crusts observed between the digital pads. (**b**) Interdigital presentation characterized by purulent discharge, with the claws appearing normal.

During physical examination, the patient appeared anxious and displayed reactive behavior towards the medical team which led to handling exclusively by the owners. The body temperature was 39.3 °C, with pale pink oral and conjunctival mucosa, and a CRT of less than 2 s. No abdominal distress was noted upon palpation. The skin and coat emitted a seborrheic odor, and multiple lesions were observed on the skin surface, characterized as follows:

- exfoliative, crusted, and ulcerated cutaneous lesions with purulent secretion localized periocular (Figure 1a and Figure 2a,b), on the nasal planum (Figure 1a,b), symmetrically in the inguinal region (Figure 3a,b and Figure 4a) and on bilateral pinnae (Figure 4b);
- severe four-limb pododermatitis characterized by exfoliation of the plantar pads accompanied by adjacent purulent secretion (Figure 5a) and interdigital pustules with associated secretion (Figure 5b).

Multiple deep skin scrapings were performed, yielding negative results, thereby excluding parasite infestation. Skin and ear cytology revealed the abundant presence of neutrophils (1–3 neutrophils/100× objective field for both ear swabs and 5–10 neutrophils/100× objective field for the skin cytology) and cocci (5–10 extracellular cocci/100× objective field for both ear swabs, 1–5 intracellular cocci/100× objective field for both ear swabs, >10 extracellular cocci/100× objective field of skin cytology, and 5–10 intracellular cocci/100× objective field of skin cytology).

Blood samples were collected for a complete blood count and serum biochemistry analysis. The complete blood count performed using Abaxis VetScan HM5 (Zoetis Services, Parsippany, NJ, USA) showed platelet counts exceeding the upper limit of the reference range, while lymphocyte (LYM), mean corpuscular hemoglobin (MCH), and mean corpuscular volume (MCV) values were at the lower end of the reference range. All of the other parameters were in normal range (Figure S1).

Serum biochemistry analysis performed using Abaxis VetScan VS2 (Zoetis Services, Parsippany, NJ, USA)—VetScan Comprehensive Diagnostic Profile indicated mild hepatic damage, with elevated levels of alkaline phosphatase (ALP) at 281 U/L and alanine aminotransferase (ALT) at 133 U/L, as well as glucose (GLU) at 115 mg/dL and potassium (K+) at 5.9 mmol/L, with all other parameters falling within normal range limits (Figure S2).

Given the exclusion of parasitic infestation, the presentation pattern of the skin lesions, and the findings from the complete blood count and serum biochemistry analysis, an abdominal ultrasound was performed, using the General Electric LOGIQ V5 Expert Ultrasound Machine (GE Medical Systems, Wuxi, Jiangsu, China) equipped with a 7.5–10 MHz microconvex transducer and a 7–13 MHz linear transducer. The abdomen was examined in a counter clockwise direction starting from the urinary bladder.

The urinary bladder appeared mildly distended with anechogenic content and normal wall layering, though thickened (0.36 cm), and presenting a slightly irregular luminal lining, with ultrasonographic findings being suggestive for cystitis.

Both kidneys presented with normal echostructure, echogenicity and size, smooth outline, and no visible cystic/nodular structures or calculi, but there were multiple calyceal and cortical punctiform hyperattenuating foci observed, which were believed to be mineralization in absence of a histopathological diagnosis (Figure S3).

The stomach had a normal wall layering and measurement with reduced content and a hyperreflective structure with a posterior acoustic shadowing-compatible with bone ingestion which was confirmed by the owner (Figure S4). The small intestine had normal wall layering and measurements and normal peristaltic movement and the large intestine presented with normal wall layering but slightly thickened (0.32 cm). The pancreas appeared normal, with no adjacent mesenteric reaction or free fluid.

The spleen appeared with predominantly normal echostructure and echogenicity, normal size (1.17 cm short axis at the level of the splenic hilum). Cranial to the splenic hilum there was a poorly delimited area, approximately 0.75 × 0.42 cm in size, non-homogeneous, showing both hyperechoic and anechoic regions, with an absence of Doppler signal, discretely deforming the splenic contour. Cranial to these there were found two other poorly defined areas, hyperechoic compared to the rest of the parenchyma, the largest measuring 0.33 × 0.25 cm. The ultrasonographic differential diagnosis established was lipomas/myelolipomas/neoplasia and focal areas of dystrophic mineralization/neoplasia (Figure S5).

**Figure 6:**
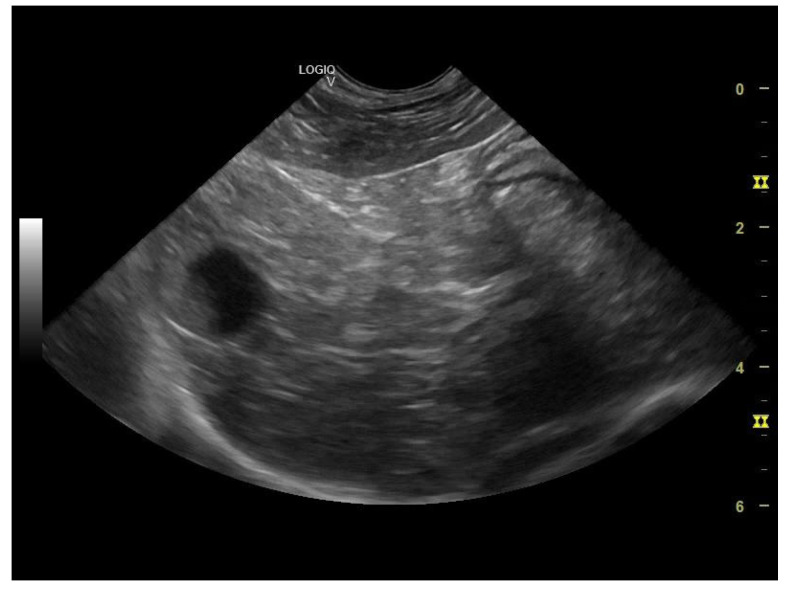
Liver and gallbladder—multiple hyperechogenic strands diffusely dispersed throughout the parenchyma—“Swiss cheese” appearance and gallbladder sediment (microconvex transducer).

**Figure 7:**
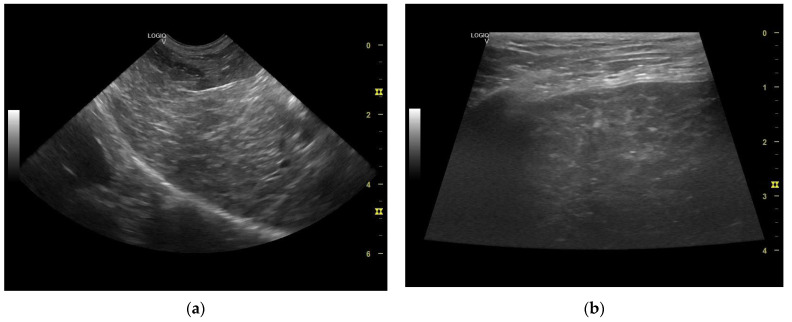
(**a**) Liver—non-homogeneous parenchyma (microconvex transducer). (**b**) Liver multiple hyperechogenic strands diffusely dispersed throughout the parenchyma—“Swiss cheese” appearance (linear transducer).

The liver appeared of normal size with non-homogeneous parenchyma, with multiple hyperechogenic strands diffusely dispersed throughout the parenchyma giving it a “Swiss cheese” appearance, with a slightly irregular outline and poorly distinguishable vascular walls (Figure 6 and Figure 7a,b). The gall bladder had a fine hyperechoic wall with anechoic content accompanied by hypoechoic, mobile, gravitationally dependent sediment in reduced quantity.

The histopathological examination of the skin and liver was proposed to the owners, but they declined the fine needle aspiration biopsy of the liver and the punch biopsy of the skin, considering the invasiveness of both sampling techniques and the need for anesthesia. The owners also declined any other parenteral treatment, requesting a protocol that could be administered by them at home. The recommended treatment consisted of a liquid oral supplement of amino acids (Rx-amino B-plex, RX Vitamins Inc., Suite, TX, USA—2 mL twice a day for 30 days), an essential fatty acid, vitamin, and zinc supplement (VetoSkin 300 mg twist off, Vetexpert, Łomianki, Poland—1 capsule/day for 30 days) and a hepatic support supplement (FOR Liver, Crida Pharm, Bucharest, Romania—1/2 pill/day for 30 days). The recommended diet consisted of a homecooked protein source (egg whites—one/day) and a commercial hepatic diet (Hill’s I/D Liver Care, Hill’s, Topeka, KS, USA). A 4% chlorhexidine shampoo (Clorhexyderm ICF, PetMart, Bucharest, Romania—2 baths/week for 4 weeks) and an ointment containing a combination of antibiotics (tetracycline, erythromycin, neomycin) and prednisolone (Mibazon, Antibiotice, Iași, Romania) one application daily for 28 days) were prescribed for the topical treatment of the pyoderma.

## 3. Discussion

This case report suggests that in dogs with characteristic skin lesions, a comprehensive diagnostic approach including abdominal ultrasound assessment of the liver and pancreas, as well as complete blood count and serum biochemistry, allows for a noninvasive diagnosis of hepatocutaneous syndrome, particularly pertinent in cases where the owners decline further invasive paraclinical examinations.

The dermatological differential diagnosis of hepatocutaneous syndrome includes autoimmune skin diseases (pemphigus foliaceus or vulgaris, systemic lupus erythematosus), drug eruption, and zinc-responsive dermatosis [3,5] as the pattern of the skin lesions could be similar. The drug cutaneous eruption diagnosis was excluded since the anamnesis did not highlight any intake of drugs that could trigger a reaction. Since zinc-responsive dermatitis develops mostly in puppies [11] as an improper feeding consequence, it was also excluded from the differential list.

Pemphigus complex is a group of autoimmune skin disease that are characterized by blister and pustule formation due to the loss of adhesion between the keratinocytes [3,5,12,13,14]. In pemphigus foliaceus, the lesions are pustular at the beginning and by pustule breaking, crusts and pyoderma are formed. The location of skin lesions in dogs are reported as follows: trunk, inner pinnae, dorsal muzzle, foot pads, periocular area, outer pinnae, planum nasale, interdigital area, lips, perianal area, and mucous membranes [14]. The pattern of the lesions is described as bilateral and symmetrical [13]. Performing direct cytology from the pustules can offer a quick diagnosis of pemphigus foliaceus if acantholytic cells are found [14]. The dog from our case report displayed crusted lesions in which acantholytic cells were not present, but a final diagnosis could not be established by dermatological examination and direct cytology alone. Pemphigus vulgaris is an autoimmune blistering dermatological pathology that especially affects the mucosae and mucocutaneous junction and can affect also the skin. The lesions evolve from blisters (vesicle or bullae) to erosions that generate pain, especially when the development is in the oral cavity [15]. In a study that corroborated the data from 54 cases of canine pemphigus vulgaris, it was concluded that the majority of cases displayed a mucosal or mucocutaneous phenotype in which the lesions were located on the lips/oral cavity. Only a few cases displayed skin lesions alone (2 out of 54) [15].

The clinical presentation of cutaneous lupus erythematosus is highly variable, ranging from minor, mild alopecic scarring lesions to extensive ulceration. Some patients exhibit pruritic seborrheic dermatitis, while others present with mucocutaneous ulceration, which can disseminate to other body regions. Depigmentation of the nose or periocular area may occur, alongside erythema, ulceration, and crusting of the nose, which is characteristic of discoid lupus erythematosus. Focal ulceration of the footpads, suggestive of vasculitis, may also be present, along with more diffuse crusting pad lesions [3].

Since the pathogenesis of the pemphigus complex diseases is related only to the immune mediated conflict at the skin/mucosal level, even if the location of the skin lesions are similar to those from pemphigus foliaceus and lupus erythematosus, in the present case report, the involvement of the liver lesions is definitory in orientating the diagnosis towards hepatocutaneous syndrome.

Previously reported abnormal clinical pathology findings in dogs diagnosed with superficial necrolytic dermatitis or hepatocutaneous syndrome include anemia, microcytosis, elevated alkaline phosphatase activity, and hypoalbuminemia. Elevated alkaline phosphatase activity was consistently noted in cases of aminoaciduric canine hypoaminoacidemic hepatopathy syndrome (ACHES), irrespective of the presence of cutaneous lesions. Anemia and microcytosis were particularly pronounced in dogs with fulminant ACHES, indicating a potential correlation between superficial necrolytic dermatitis and these hematological alterations. Profiling of plasma and urine amino acids offers a noninvasive approach for disease confirmation. As previously demonstrated, urine amino acid profiling normalized to creatinine, revealing lysinuria, presents a novel diagnostic parameter that could enhance the diagnostic efficacy of plasma amino-acid profiling [16].

The patient in this study exhibited subtle modifications in the complete blood count, specifically lymphocytes (LYM), mean corpuscular volume (MCV), and mean corpuscular hemoglobin (MCH) at the lower limit of the reference range, while platelets (PLT) exceeded the upper limit of the reference range, thus suggesting the presence of microcytic anemia. Serum biochemistry indicated values above the limit for alkaline phosphatase (ALP) and alanine aminotransferase (ALT), with albumin levels within physiological limits. A limitation of this case report is the inability to quantitatively determine plasma and urinary amino acid values due to external reasons (infrastructure liabilities of the laboratory and the lack of other options in the geographical area).

The ultrasonographical aspect of the liver as well as the normal size of the organ combined with the Swiss cheese-like appearance of the parenchyma corroborates the diagnosis of hepatocutaneous syndrome. Another differential diagnosis in absence of the dermatological findings could have been that of chronic hepatitis/liver cirrhosis, although the liver is reported to be reduced in size in these common conditions.

Historically, superior outcomes for dogs with hepatocutaneous syndrome have been attributed to the administration of intravenous amino acid solutions [17]. Alongside intravenous amino acid infusions, high-protein diets, typically commercial diets supplemented with whey protein, have been a cornerstone of treatment for dogs with hepatocutaneous syndrome [18]. More recently, the combined administration of intravenous lipid with intravenous amino acid infusions was reported to manage hepatocutaneous syndrome in a single dog for 24 months. These observations suggest improved remission and survival in dogs with aminoaciduric canine hypoaminoacidemic hepatopathy syndrome when fed high-protein home-cooked diets [2]. In a separate study, the utilization of a combined treatment approach involving amino acid supplementation and stem cell therapy demonstrated efficacy in a patient diagnosed with hepatocutaneous syndrome. Notably, the dog exhibited survival for a period of 32 months following diagnosis [19].

In the case report presented, the owner of the dog requested a treatment protocol that could be administered at home. The treatment protocol included oral supplements of amino acids, minerals and vitamins, essential fatty acids, and a hepatic support supplement, as well as a commercial hepatic diet supplemented with a home-cooked protein source. Additionally, topical treatment of the pyoderma was prescribed, consisting of using twice a week a 4% chlorhexidine shampoo and an ointment for daily use, containing a combination of antibiotics (tetracycline, erythromycin, neomycin) and an anti-inflammatory (prednisolone). However, the follow-up of the patient could not be conducted as the owner did not attend the 30-day recheck appointment.

The majority of reported outcomes for dogs with hepatocutaneous syndrome are poor, with an average survival of 3–6 months from the time of diagnosis [4,20,21], although sporadic reports indicate survival times exceeding 2 years [2].

It has been hypothesized that dermal lesions manifest as an advanced phase of hepatocutaneous syndrome, and their absence, consequently, does not exclude the presence of the condition [16]. Hence, particularly when dermatologic patients exhibit cutaneous symptoms suggestive of hepatocutaneous syndrome, it is assumed that abdominal ultrasound evaluation serves as an essential non-invasive diagnostic tool and therefore, it was considered that in this situation the histopathological examination of the liver would not be mandatory, once ultrasonographic aspects of the liver are considered pathognomonic [9,22].

## 4. Conclusions

The hepatocutaneous syndrome of the dog appears to be a complex pathology that can be easily confused with other skin diseases such as pemphigus complex skin diseases, lupus erythematosus, zinc responsive dermatitis, or drug-induced cutaneous reaction and for whose diagnosis the paraclinical examinations are of particular importance. A non-invasive approach of diagnosing the hepatocutaneous syndrome in a dog by clinical examination, routine blood testing, and ultrasound assessment of the abdomen was pursued in the absence of the possibility of performing a histopathological diagnosis.

Ultrasound examination of the abdomen is not a frequently used paraclinical examination technique for the dermatological patients, and it should be considered before other options. We conclude that in patients with clinical symptoms compatible with the previously mentioned pathologies, the first step in establishing a diagnosis should be the exclusion of hepatocutaneous syndrome by non-invasive methods, and unless liver damage is excluded, histopathological examination of the skin should be considered necessary.

## Data Availability

The data present in this study are available within the article.

## Supplementary Materials

The following supporting information can be downloaded at: https://www.mdpi.com/article/10.3390/life14070853/s1. Figure S1: Complete blood count of the patientt; Figure S2: Biochemistry serum analyses of the patient; Figure S3: Spleen- hyperechoic marginal areas (linear transducer); Figure S4: Stomach- hyperreflective structure with a posterior acoustic shadowing (microconvex transducer); Figure S5: Kidney- hyperechoic cortical foci (linear transducer).
