# Supplementary figures and images for: Non-Invasive Paraclinical Diagnosis of Hepatocutaneous Syndrome in a Dog
Source: Life (Basel). 2024 Jul 8;14(7):853. doi: 10.3390/life14070853 (PMC11277621; doi:10.3390/life14070853)

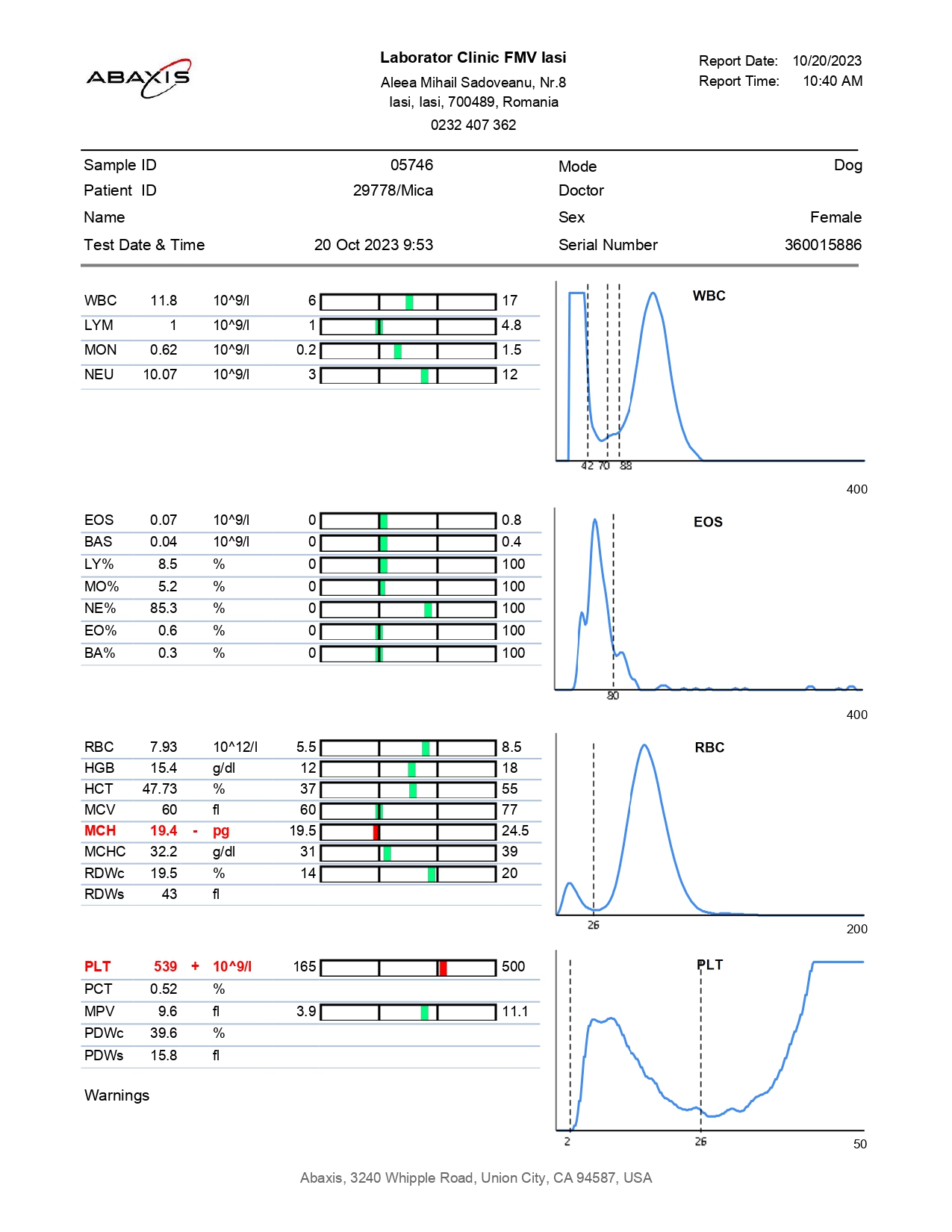

Supplement: Supplementary file 1 [file life-14-00853-s001.zip › Figure S1 Complete blood count of the patient.jpeg]

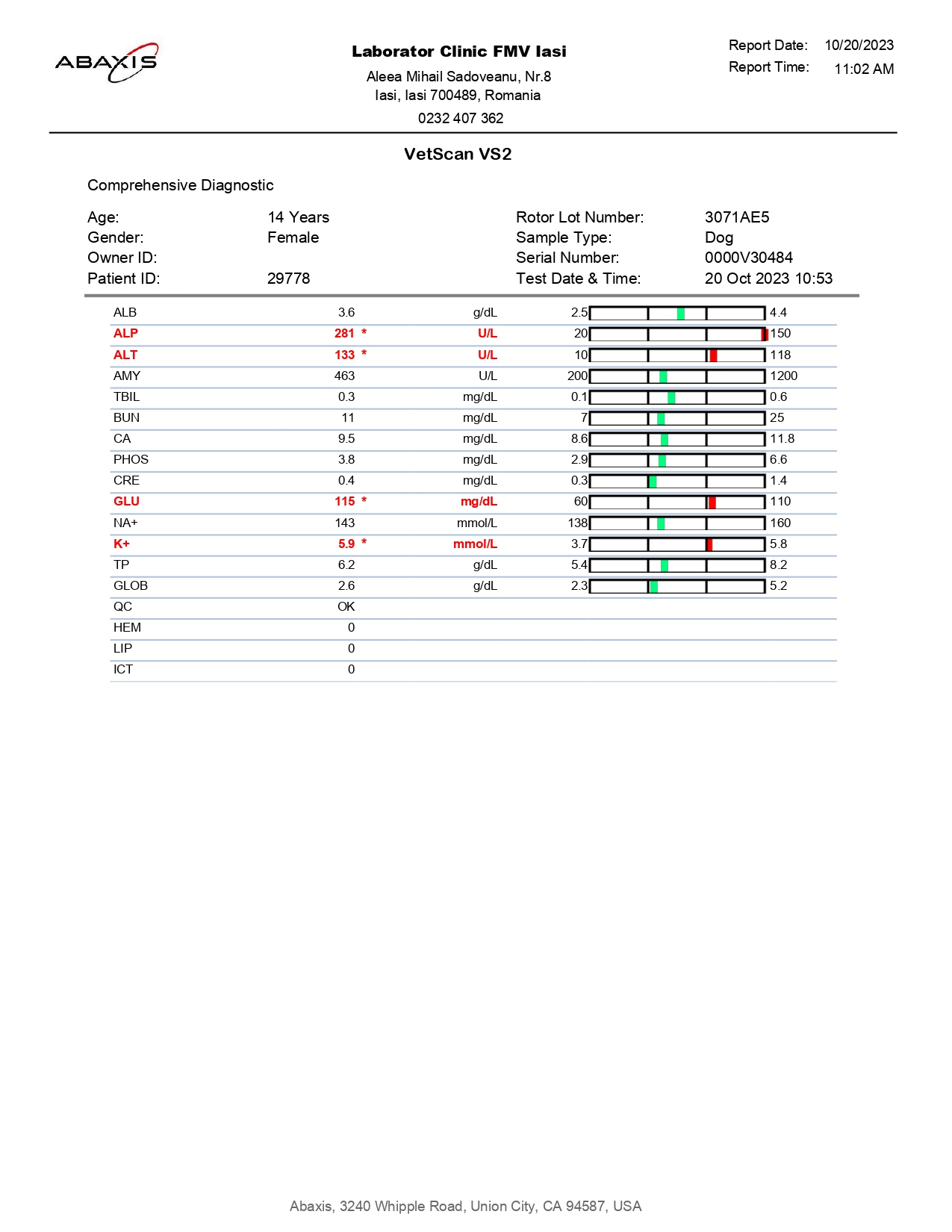

Supplement: Supplementary file 1 [file life-14-00853-s001.zip › Figure S2. Biochemistry serum analyses of the patient.jpeg]

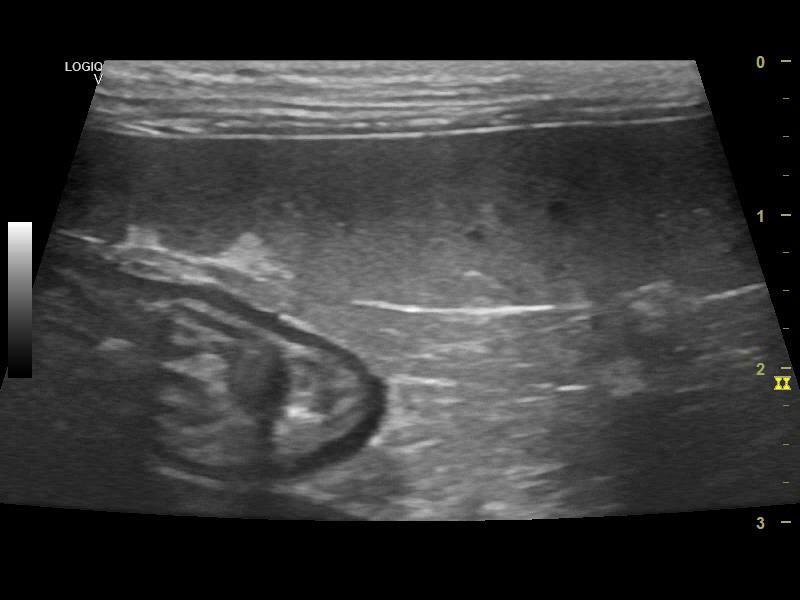

Supplement: Supplementary file 1 [file life-14-00853-s001.zip › Figure S3 Spleen- hyperechoic marginal areas (linear transducer).jpg]

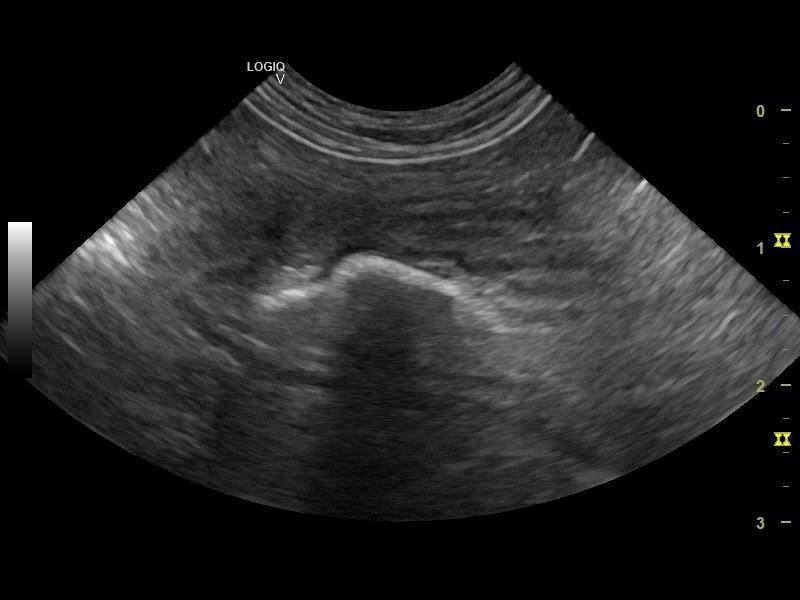

Supplement: Supplementary file 1 [file life-14-00853-s001.zip › Figure S4. Stomach- hyperreflective structure with a posterior acoustic shadowing (microconvex transducer).jpg]

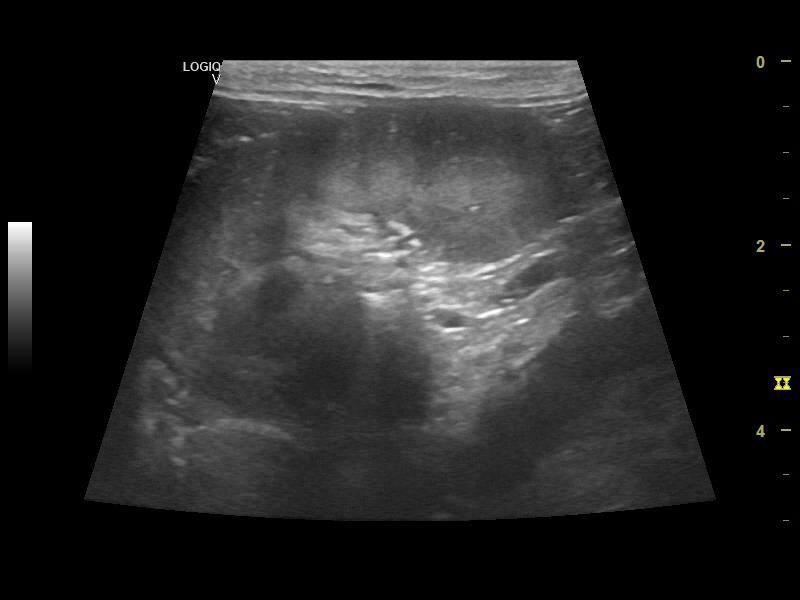

Supplement: Supplementary file 1 [file life-14-00853-s001.zip › Figure S5-Kidney- hyperechoic cortical foci (linear transducer).jpg]
